# The nucleotide specificity of succinyl‐CoA synthetase of *Plasmodium falciparum* is not determined by charged gatekeeper residues alone

**DOI:** 10.1002/2211-5463.13034

**Published:** 2021-02-17

**Authors:** Kapil Vashisht, Pallavi Singh, Sonia Verma, Rajnikant Dixit, Neelima Mishra, Kailash C. Pandey

**Affiliations:** ^1^ Protein Biochemistry and Engineering Lab, Parasite‐Host Biology Group ICMR–National Institute of Malaria Research New Delhi India

**Keywords:** gatekeeper residues, malaria, *Plasmodium falciparum*, site‐directed mutagenesis, substrate specificity, succinyl‐CoA synthetase

## Abstract

Substrate specificity of an enzyme is an important characteristic of its mechanism of action. Investigation of the nucleotide specificity of *Plasmodium falciparum* succinyl‐CoA synthetase (SCS; *Pf*SCS) would provide crucial insights of its substrate recognition. Charged gatekeeper residues have been shown to alter the substrate specificity via electrostatic interactions with approaching substrates. The enzyme kinetics of recombinant *Pf*SCS (wild‐type), generated by refolding of the individual *P. falciparum* SCSβ and *Blastocystis* SCSα subunits, demonstrated ADP‐forming activity (*K*_mATP_ = 48 µm). Further, the introduction of charged gatekeeper residues, either positive (Lys and Lys) or negative (Glu and Asp), resulted in significant reductions in the ATP affinity of *Pf*SCS. It is interesting to note that the recombinant *Pf*SCSβ subunit can be refolded to a functional enzyme conformation using *Blastocystis* SCSα, indicating the possibility of subunits swapping among different organisms. These results concluded that electrostatic interactions at the gatekeeper region alone are insufficient to alter the substrate specificity of *Pf*SCS, and further structural analysis with a particular focus on binding site architecture is required.

## Graphical abstract

Electrostatic interactions between charged gatekeeper residues of the enzyme and approaching substrates can effectively discriminate between similar substrates. We investigated whether the charged gatekeeper residues play any role in the substrate specificity of *P. falciparum* succinyl‐CoA synthetase (*Pf*SCS). Our results demonstrated that charged gatekeeper residues in *Pf*SCS did alter the ATP binding but could not alter its substrate specificity, pointing toward other factors affecting the substate specificity of *Pf*SCS.

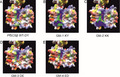

**Abbreviations**
IB: inclusion body
MSA: multiple sequence alignment
PDB: Protein Data Bank
*Pf*SCS: *Plasmodium falciparum* succinyl‐CoA synthetase
SCS: succinyl‐CoA synthetase
TCA: tricarboxylic acid
WT: wild‐type

In any biological system, the substrate specificity is a characteristic property of the enzymes. There are two landmark models to describe substrate specificity of an enzyme: ‘lock‐and‐key model’ [1] proposes a rigid fit, whereas the ‘induced fit model’ [2] suggests a flexible nature of the enzyme to fit the substrate. At the molecular level, the substrate specificity is best described by the molecular interactions of a protein and its substrates. The free energies of the hydrogen bonds between a protein–substrate and the propensity of specific amino acids around the substrate play a critical role in determining the substrate specificity of an enzyme [3]. In addition, weak interactions, such as van der Waals and electrostatic interactions [4], between the protein and its substrate also have significant contribution in the substrate specificity of an enzyme, especially when the proteins have to discriminate between two similar substrates, e.g. adenine and guanine, in the case of nucleotide‐binding proteins. Basu *et al*. [4] reported that a strong ligand‐free electrostatic potential could discriminate between A/G binding sites, and hence established the role of an electrostatic component in the molecular discrimination of adenine and guanine. Previously, the electrostatic potential arising from the charged amino acids inside the active site of the subtilisin enzyme had been shown to be functionally significant [5]. However, the role of other charged amino acids near or outside the active site has not been investigated thoroughly. In 2008, Hamblin et al. [6] proposed an electrostatic gatekeeper effect, in which the nucleotide access was controlled by the charged amino acids (gatekeeper residues) outside the binding site of the succinyl‐CoA synthetase (SCS) of *Blastocystis*, a human intestinal parasite. Recently, we have experimentally demonstrated the ‘electrostatic gatekeeper effect’, where the gatekeeper residues were found to be critical for nucleotide specificity in *Blastocystis* SCS [7]. Interestingly, this study also established a novel enzyme engineering approach, where the switching of the charge of the gatekeeper residues from positive to negative demonstrated that the ADP‐forming SCS could also utilize GTP. Surprisingly, two binding site modifications in addition to the charge switching resulted in a complete reversal of an ADP‐forming SCS to GDP‐forming SCS.

To further signify the role of gatekeeper residues in determining the nucleotide specificity, we explored another model enzyme, SCS of *Plasmodium falciparum*. *P. falciparum* is an important human parasite that causes malaria, a significant infectious disease, with ~219 million clinical cases and ~0.43 million deaths worldwide (https://www.mmv.org/newsroom/publications/world‐malaria‐report‐2018). The first line of defense for *P. falciparum* malaria is artemisinin combination therapies. However, the emergence of resistance against artemisinin combination therapies is a matter of great concern, as was with the previous generation of antimalarials, such as chloroquine, sulfadoxine and pyrimethamine. Therefore, a considerable amount of effort is currently being devoted to identify novel drug targets for malaria, simultaneously expanding the fundamental understanding of *Plasmodium* biology. SCS is a crucial enzyme of the tricarboxylic acid (TCA) cycle, for its unique capability of generating ATP via substrate‐level phosphorylation. In *P. falciparum*, however, the TCA cycle has been suggested to be of limited importance [8], yet the parasite synthesizes all the TCA cycle enzymes [9]. During the asexual growth of the malaria parasite, the absence of any specific phenotypes in *ΔKDH/ΔSCS* and *ΔSCS/ΔSDH* knockout lines (KOs), indicated metabolic plasticity in the TCA cycle (where KDH represents α‐ketoglutarate dehydrogenase, SCSα subunit, and SDH represents SDH flavoprotein subunit) [10]. Unlike the asexual stages of *P. falciparum*, the SCS is significant in terms of maintaining the reserves of succinyl‐CoA, as an initial substrate for heme biosynthesis along with glycine for its sexual stages [11]. This study explored the alteration of the charge of the gatekeeper residues and its subsequent effect on the substrate specificity of *Pf*SCS.

## Materials and methods

### Computational analysis of the SCS subunits

SCS is composed of two subunits, SCSα and SCSβ, whereas the SCSβ subunit carries the only nucleotide binding site. The amino acid sequences of the SCSβ subunits from phylogenetically diverse organisms were retrieved from UniProtKB, and respective details are summarized in Table 1. A multiple sequence alignment (MSA) of these sequences was performed using ClustalO. The alignment output representation was performed by Boxshade server. Weblogos were also generated from the respective alignments of the ADP‐forming and GDP‐forming SCS to identify the most frequently present gatekeeper residues. After identification of the gatekeeper residues from the MSA, various mutants were designed in an attempt to alter the charge of the gatekeeper residues; details are summarized in Table 2. Structure models were generated for the wild‐type (WT) and various mutant *Pf*SCSβ subunits by using modeler 9v13 (University of California San Francisco, CA, USA), with the following templates, *E. coli* SCS [Protein Data Bank (PDB): 1CQI] [12] and pig SCS (PDB: 2FP4) [13]. The models were further analyzed by Ramachandran scatterplots and DOPE scores. The electrostatic surfaces of the gatekeeper regions were also constructed using eF‐surf server and visualized using PDBjViewer [14].

**Table 1:**
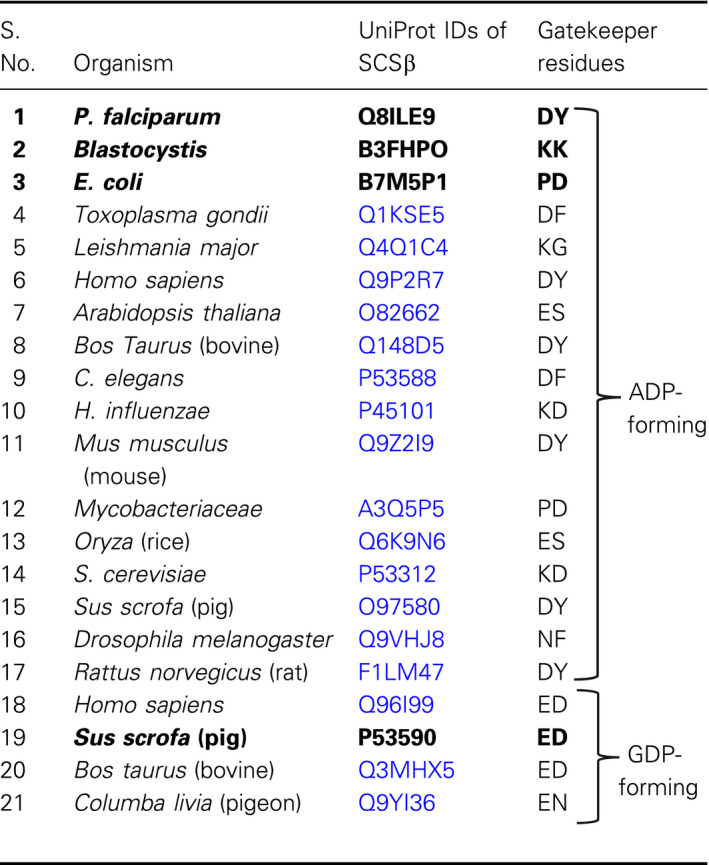
List of ADP‐forming and GDP‐forming SCSβ subunits from various organisms with their UniProt IDs. The gatekeeper residues in bold and shaded rows have been used for comparison in previous studies also [7].

**Table 2:** Nomenclature of the WT and various gatekeeper mutants of *Pf*SCSβ subunits and the respective primer sequences used for cloning. Restriction enzymes are italicized and underlined for the WT *Pf*SCSβ subunit. Mutations are represented by underlined lowercase nucleotides (bold and italics) in the respective mutants. FP, forward primer; RP, reverse primer.

Gatekeeper residues of *Pf* SCSβ (bold and italics)	Mutations	Primers
*Pf* SCSβ WT ***DY*** (WT‐DY)	No mutation	FP: 5′‐TAT*GGATCC*ATGGCCCGTTTTAAGAGCC‐3′ [*BamHI*] RP: 5′‐ATT*GTCGAC*TTAAACGAGATGTCTATG‐3′ [*SalI*]
Gatekeeper mutant‐1 ***KY*** (GM‐1 KY)	***D*** →***K*** at 95 position	FP: 5′‐TGGTGATAAT*aag*TTAGTAATAAAAGCTC‐3′ RP: 5′‐CAAACGTTTTGTAATAATAAAGC‐3′
Gatekeeper mutant‐2 ***KK*** (GM‐2 KK)	***Y*** →***K*** at 164 position	FP: 5′‐GAACGTTTTT*aag*TAAGAAAAGAAAGATATATTGC‐3′ RP: 5′‐ACATATAAATACAGTATTACATTTTTTTC‐3′
Gatekeeper mutant‐3 ***DE*** (GM‐3 DE)	***Y*** →***E*** at 164 position	FP: 5′‐GAACGTTTTT*gag*TAAGAAAAGAAAGATATATTGCTTTTC‐3′ RP: 5′‐ACATATAAATACAGTATTACATTTTTTTC‐3′
Gatekeeper mutant‐4 ***ED*** (GM‐4 ED)	***D*** →***E*** at 95 and ***Y***→***D*** at 164 positions, respectively	FP: 5′‐TGGTGATAAT*gag*TTAGTAATAAAAG‐3′ RP: 5′‐CAAACGTTTTGTAATAATAAAGC‐3′

### Determination of the nucleotide specificity of native *Pf*SCS enzyme

The nucleotide specificity of the native *Pf*SCS enzyme was determined from the lysate of the cultured *P. falciparum* strain 3D7, as described earlier [15]. In brief, the parasites were grown in human erythrocytes using 2% hematocrit in RPMI‐1640 supplemented with 10% human serum. The lysate was prepared by saponin lysis and ultrasonication of the cultured parasites, centrifuged at 25,000 *g* for 15 min at 4 °C. The supernatant was collected, and enzymatic assays were performed as described earlier [6]. In brief, the supernatant containing the native *P. falciparum* SCS was added to the assay buffer [129 μm CoA, 10 mm sodium succinate, 50 mm KCl, 10 mm MgCl_2_ and 50 mm Tris–HCl (pH 7.4)] with respective nucleotide substrates (ATP and GTP, 150 µm each). The assay recorded the formation of a thioester bond in succinyl‐CoA at 232 nm.

### Cloning, recombinant protein expression and refolding of *Pf*SCS

The *Pf*SCSβ WT subunit was amplified using the primer sequences given in Table 2. The amplified *Pf*SCSβ gene was ligated in expression vector pET28a vector (Novagen, Merck KGaA, Darmstadt, Germany) with 6X His‐tag, using appropriate restriction sites and transformed into *E. coli* (DH5α cells). For recombinant protein expression, the *Pf*SCSβ + pET28a construct was transformed into *E. coli* BL21‐CodonPlus® competent cells. The *Pf*SCSβ gatekeeper mutants were generated by a commercially available Q5 site‐directed mutagenesis kit (New England Biolabs, MA, USA) and confirmed by sequencing of the constructs for desired mutations at respective positions. The respective primer sequences for substituting the codons are mentioned in Table 2. Despite multiple efforts, it was not possible to clone the *Pf*SCSα subunit; hence the *Blastocystis* SCSα subunit (having >60% identity with *Pf*SCSα) was chosen to generate the refolded *Pf*SCS enzyme.

The protein expression was carried out using standard protocols, optimized in the laboratory [7]. In brief, the overexpression of the cloned *Pf*SCSβ subunit was induced by the addition of 1 mm IPTG after the *A* values reached 0.4–0.6, and was grown for 4 h postinduction. The bacterial cell pellets were reconstituted in lysis buffer [50 mm NaH_2_PO_4_, 10 mm Tris, 500 mm NaCl, 10 mm imidazole (pH 8.0)] and sonicated. Centrifugation at 25,000 *g* for 30 min at 4 °C yielded the supernatant and cell debris pellet. The pellet was further processed for isolation of inclusion bodies (IBs) containing the 6X‐His‐tagged *Pf*SCSβ subunits, washed twice with 1M urea and 1% Triton X‐100, and finally with 1 m urea alone. The IBs were solubilized in solubilization buffer containing 6 m guanidine hydrochloride and 10 mm Tris–HCl (pH 8.0) overnight. The purification of *Pf*SCSβ was carried out by a custom‐packed column with Ni‐NTA resin (Nucleopore; Genetix Biotech Asia, Delhi, India) using a fast‐process liquid chromatography system, AKTA Prime, FPLC (GE Life Sciences, MA, USA). The elutions were collected from the 200 mm imidazole fractions and analyzed by SDS/PAGE. The *Pf*SCSβ subunit was confirmed by western blotting using a commercially available mouse monoclonal antibody raised against 6X‐His‐tag (Sigma‐Aldrich, Merck KGaA, Darmstadt, Germany). As mentioned previously, the *Blastocystis* SCSα subunit was used at the time of refolding with *Pf*SCSβ subunit [7]. Both the subunits were again denatured in the solubilization buffer and concentrated using 10 kDa cutoff Centricons (Vivaspin). Optimized refolding was performed in buffer [50 mm Tris–HCl, 25% glycerol, 25 mm DTT and 100 µm MgCl_2_ (pH 7.2)] with rapid dilution (100‐fold) of the respective subunits in 1 : 1 ratio and incubated overnight at 4 °C. The refolded *Pf*SCS enzymes were again concentrated with a 10 kDa cutoff Amicon stirred‐cell (Millipore, Merck KGaA, Darmstadt, Germany) and centrifuged at 14 500 r.p.m. for 15 min at 4 °C, to remove precipitated/misfolded proteins, before performing the enzymatic assays.

### Enzyme kinetics of the *Pf*SCS (WT and various gatekeeper mutants)

Enzymatic assays were performed with optimized conditions in buffer [10 mm sodium succinate, 50 mm KCl, 10 mm MgCl_2_ and 50 mm Tris–HCl (pH 7.4)]. One hundred twenty‐nine micromolar CoA and ~30 nm refolded *Pf*SCS enzymes (WT and various gatekeeper mutants) were added in each reaction mix. Varying concentrations of ATP and GTP were used to carry out the enzymatic reaction. The product formation was followed for 10 min with 1‐min intervals. A UV‐absorbance at 232 nm was recorded in the quartz cuvette of 10‐mm path length corresponding to the formation of a thioester bond in succinyl‐CoA. The enzyme kinetics results were analyzed to calculate the Michaelis–Menten constant (*K*_m_) by using (graphpad prism, ca, usa) 5.0 software.

## Results

### Sequence and molecular modeling analysis of the various SCSβ subunits

**Fig. 1:**
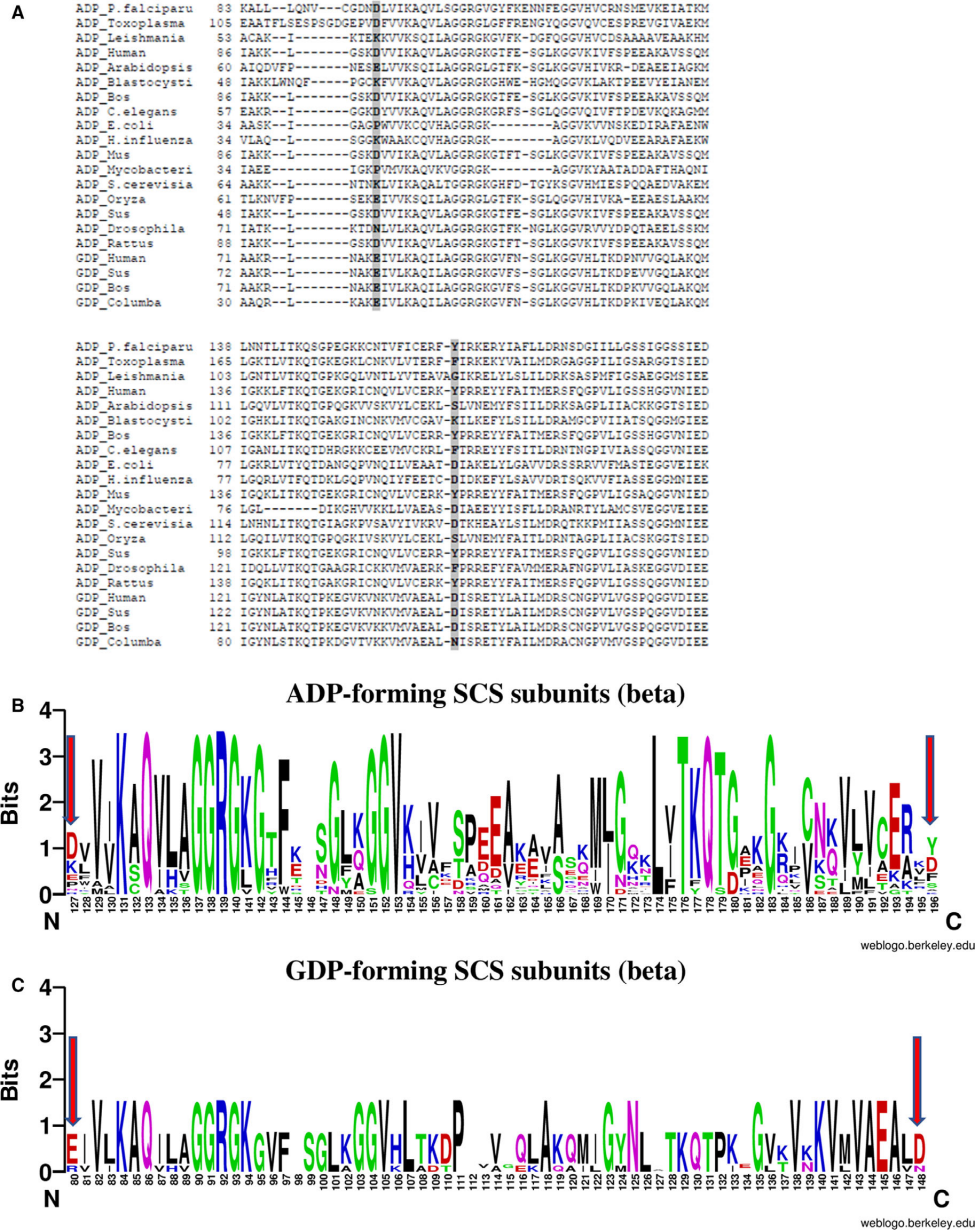
(A) MSA of various SCSβ subunits from phylogenetically diverse organisms (gatekeeper residues are shaded in gray). Weblogo representing the gatekeeper residues in (B) ADP‐forming SCSβ subunits and (C) GDP‐forming SCSβ subunits, indicating the most common residues from the representative organisms aligned in the previous figure (highlighted by red arrows). The height of the amino acid letter indicates its prevalence in the number of sequences available.

The MSA of SCSβ subunit sequences from various organisms is presented in Fig. 1A, and the respective gatekeeper residues are shaded. Among the ADP‐forming SCSβ subunits, the gatekeeper residues are listed in Table 1. Human intestinal parasite *Blastocystis* SCS has Lys and Lys (positively charged) gatekeeper residues, whereas *Pf*SCS has Asp and Tyr (negatively charged and hydrophobic) gatekeeper residues. Another apicomplexan parasite, *Toxoplasma gondii*, also has the negatively charged and hydrophobic gatekeeper residues (Asp and Phe), but *Leishmania major* has positive and nonpolar (Lys and Gly) gatekeeper residues. Two representative plant species, *Arabidopsis* and *Oryza*, have negatively charged and polar/uncharged gatekeeper residues (Glu and Ser, respectively). The ADP‐forming SCSβ subunits of *Homo sapiens*, *Bos taurus*, *Mus musculus* and *Sus scrofa* have the similar gatekeeper residues as *P. falciparum* (Asp and Tyr); however, the GDP‐forming SCSβ subunits of *H. sapiens*, *B. taurus* and *S. scrofa* have the negatively charged gatekeeper residues, Glu and Asp. The weblogos demonstrated that the most common gatekeeper residues among the ADP‐forming SCSβ subunits are Asp and Tyr (Fig. 1B), whereas in the GDP‐forming SCSβ subunits, the most frequently present gatekeeper residues are Glu and Asp (Fig. 1C). From the MSA, we have designed various gatekeeper mutants of the *Pf*SCSβ subunit, particularly to alter the charge at the gatekeeper region (Table 2).

**Fig. 2:**
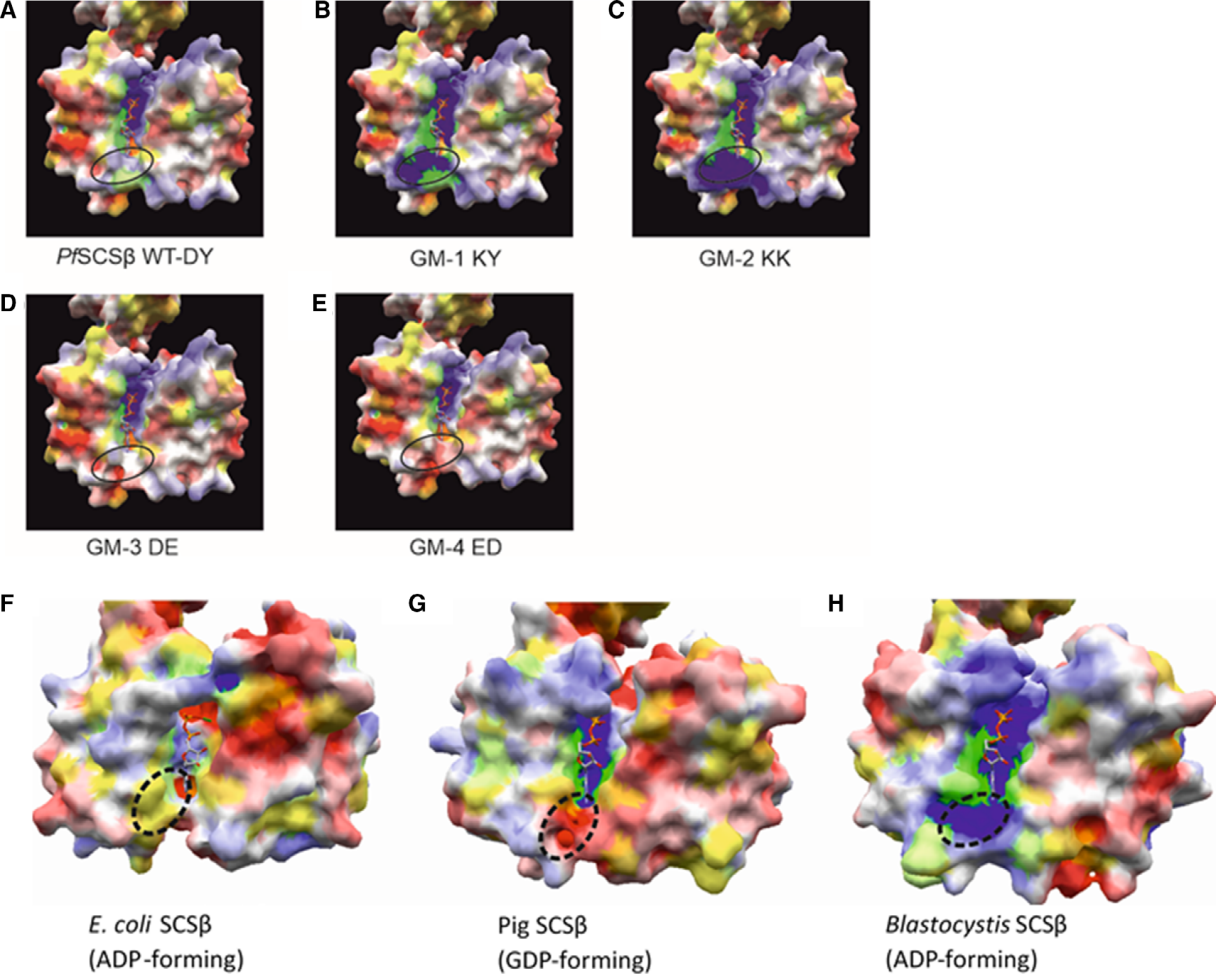
Snapshots of the electrostatic surface models of the *Pf*SCSβ gatekeeper region. Electrostatic surfaces of the gatekeeper region of *Pf*SCSβ subunits indicated by a black oval. (A) Gatekeeper region of WT‐DY. (B) Gatekeeper region of the gatekeeper mutant GM‐1 KY. (C) Gatekeeper region of GM‐2 KK. (D) Gatekeeper region of GM‐3 DE. (E) Gatekeeper region of GM‐4 ED. The electrostatic surface of the gatekeeper region shown in red indicates an overall negative charge, blue indicates positive charge and purple indicates the polar character of the residue. The electrostatic surfaces were prepared by using Modeller9V1032 and eF‐surf server and visualized in PDBjViewer. For reference, the SCSβ subunits from *E. coli*, Pig and *Blastocystis* are also represented here (F–H) [7].

The molecular models of *Pf*SCSβ subunits from WT and various gatekeeper mutants were generated, and further electrostatic surfaces were constructed for all the models. The snapshots of the gatekeeper region of the *Pf*SCSβ subunits are represented in Fig. 2. The *Pf*SCSβ WT‐DY carried the negatively charged and polar gatekeeper residues (Asp and Tyr), and hence the corresponding gatekeeper region represents the negative and polar character (Fig. 2A). *E. coli* SCSβ subunit displayed the gatekeeper region as negative and nonpolar as a result of Pro and Asp residues at the gatekeeper region (Fig. 2F). GM‐1 KY and GM‐2 KK were constructed by sequential substitutions of Asp→Lys and Tyr→Lys, respectively, which are indicated by the presence of positive charge at the gatekeeper region (Fig. 2B,C). Other gatekeeper mutants, GM‐3 DE and GM‐4 ED, both carried the negative gatekeeper residues, whereas it is only the latter that emulated the negatively charged Glu and Asp from the pig SCSβ subunit (Fig. 2D,E). Interestingly, the gatekeeper region did not show the negatively charged gatekeeper region as intense as it did in pig SCSβ (Fig. 2G) [6].

### Determination of the nucleotide specificity of native and recombinant *Pf*SCS enzymes

**Fig. 3:**
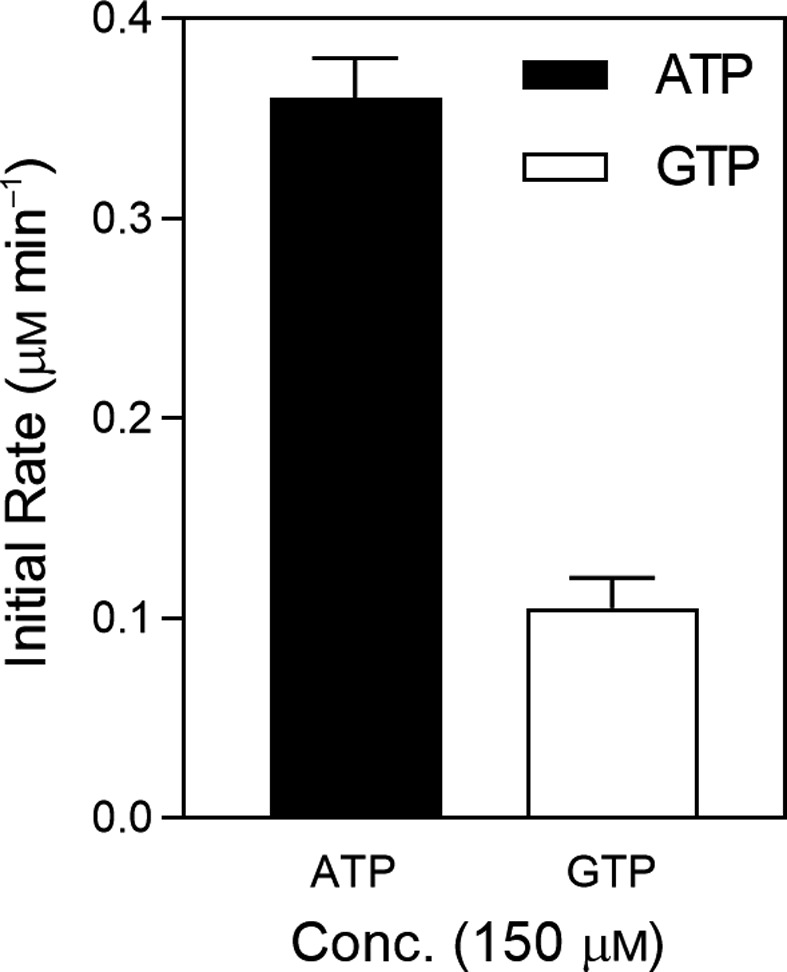
Initial rates of reaction for native *Pf*SCS enzyme. *Pf*SCS enzyme activity with both nucleotides (ATP and GTP) at 150 µm concentration (Conc.). The error bars represent the standard error of the mean from duplicate experiments.

The nucleotide specificity of *Pf*SCS was determined from the crude lysate of *in vitro*‐cultured *P. falciparum* using the enzymatic assay, as described by Hamblin *et al*. [6]. In accordance with the previous assumption, because of the presence of negative and hydrophobic gatekeeper residues of the *E. coli* SCSβ subunit, the *Pf*SCS enzyme should use both nucleotides (ATP and GTP). However, the native *Pf*SCS enzyme was found to be predominantly ADP forming, having some insignificant activity with the GTP (Fig. 3).

**Fig. 4:**
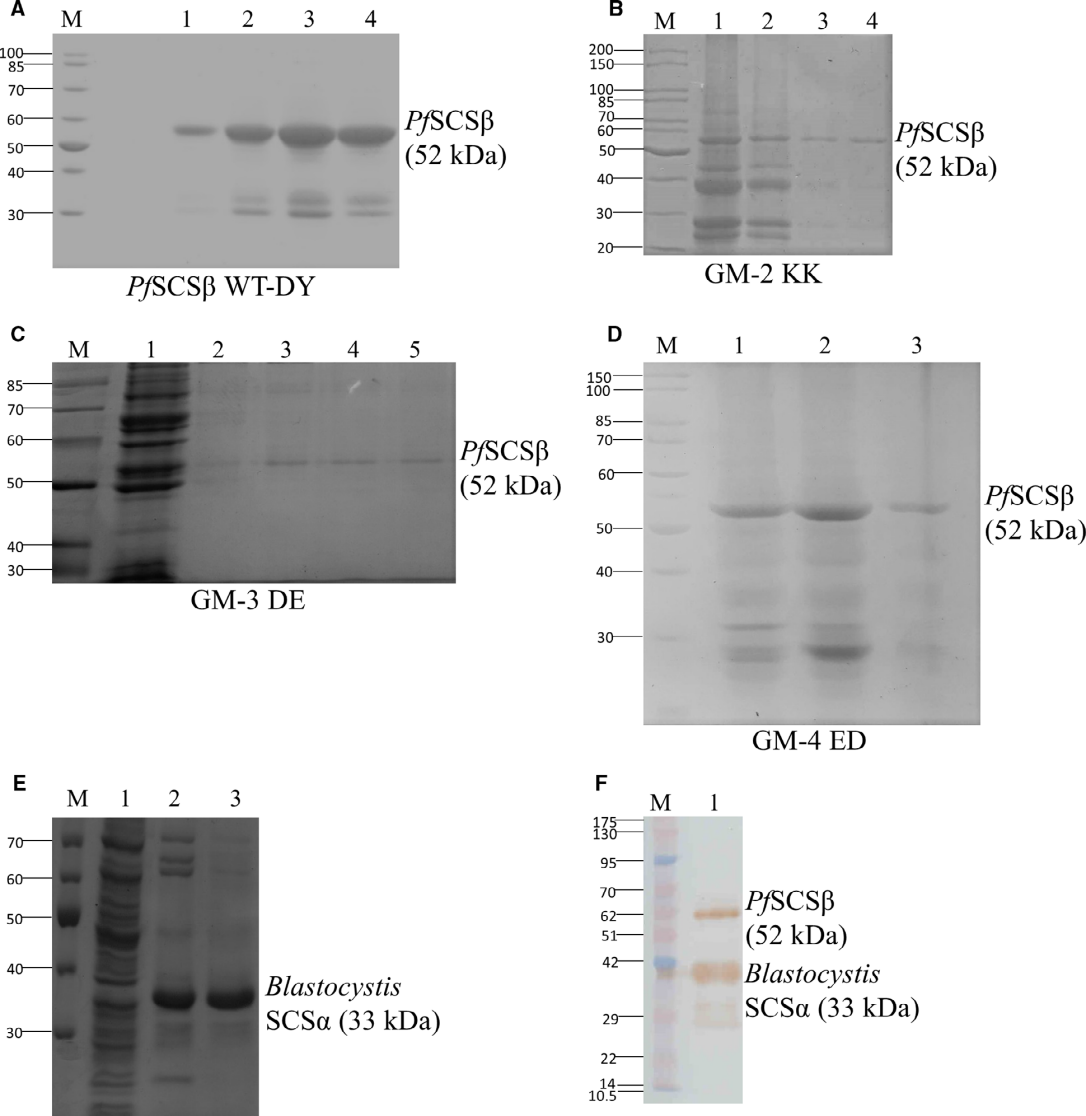
SDS/PAGE analysis of the *Blastocystis* SCSα and *Pf*SCSβ subunits. (A) *Blastocystis* SCSα, lanes 2 and 3 containing purified fractions at size 33 kDa. (B) *Pf*SCSβ WT‐DY containing purified fractions in lanes 4–6 at size 52 kDa. (C) Gatekeeper mutant GM‐2 KK containing purified fractions in lanes 3 and 4 at size 52 kDa. (D) GM‐3 DE containing purified fractions in lanes 3 and 4 at size 52 kDa. (E) GM‐4 ED containing purified fractions in lane 3 at size 52 kDa. (F) Western blot of the *Pf*SCSβ WT‐DY and *Blastocystis* SCSα subunits detected by anti‐His antibody (protein marker is represented by kDa).

Recombinant protein expression was carried out in *E. coli* (BL21DE3) cells for all the *Pf*SCSβ subunits, including the WT and its various gatekeeper mutants. The affinity chromatography‐purified fractions of *Pf*SCSβ subunits from the IBs were analyzed by SDS/PAGE (Fig. 4B–E), and as mentioned previously, the 6X‐His‐tagged *Blastocystis* SCSα was purified separately in native conditions by affinity chromatography (Fig. 4A). The *Pf*SCSβ WT‐DY and the *Blastocystis* SCSα subunits were confirmed by western blot showing the presence of two expected size bands by mouse monoclonal anti‐His antibody (Fig. 4F). Before proceeding for the enzymatic analysis of the recombinant *Pf*SCS, the WT and gatekeeper mutants were refolded as described in Materials and methods.

**Fig. 5:**
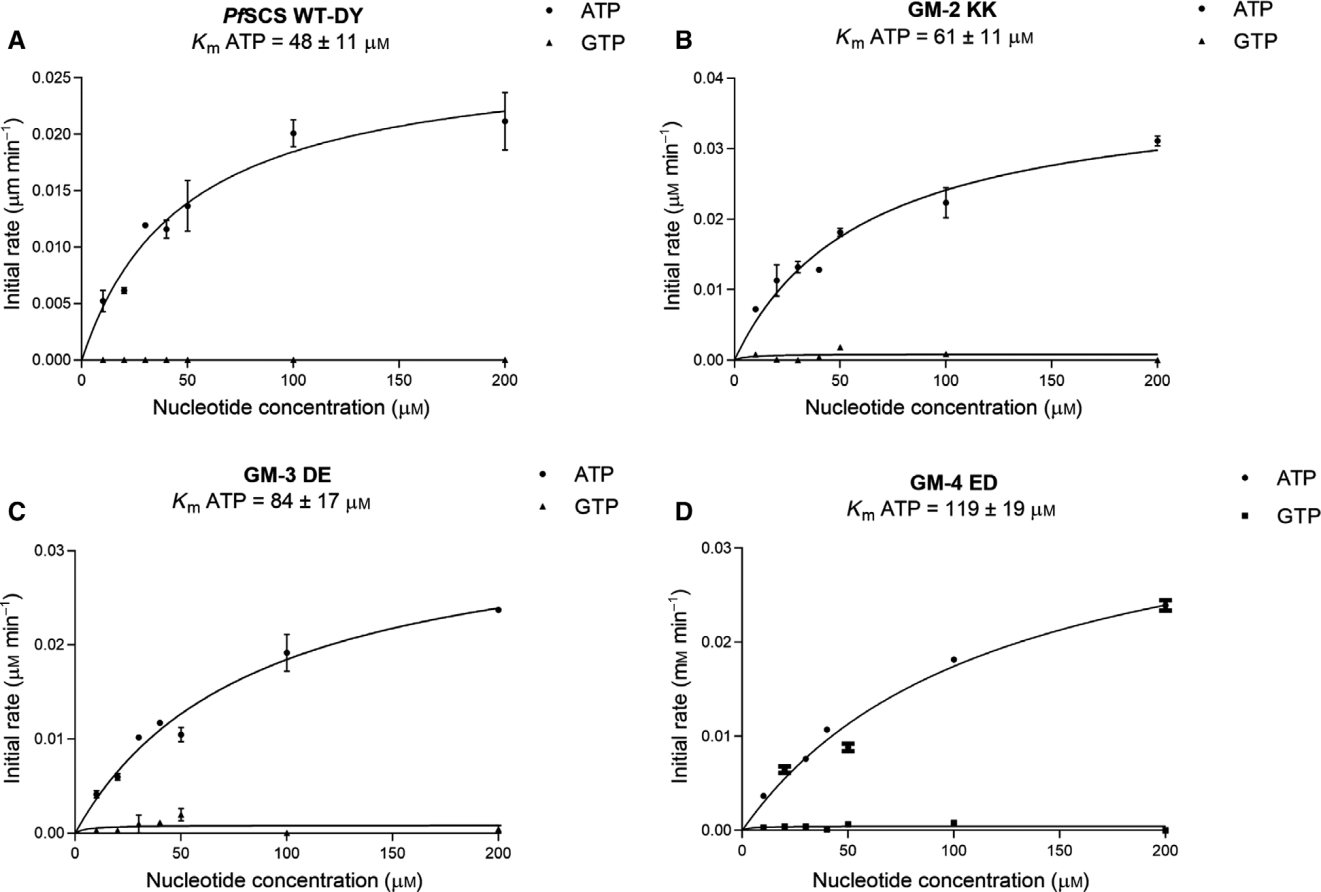
Enzyme kinetics of *Pf*SCS recombinantly expressed and refolded enzymes with variable concentrations of ATP and GTP. Graphs are showing the initial rates (µm·min^−1^) versus ATP and GTP concentrations (µm). Graph of *Pf*SCS WT‐DY (A), GM‐2 KK (B), GM‐3 DE (C) and (D) GM‐4 ED. The *K*_m_ values were calculated by graphpad prism 5.0. The error bars represent the standard error of the mean from duplicate experiments.

It is interesting to note that the *Pf*SCSβ and *Blastocystis* SCSα subunits were separately denatured and refolded into active enzyme confirmations, as per optimized protocols. Because the nucleotide‐binding site lies in the SCSβ subunit, this unique approach was followed after failed attempts to clone the *Pf*SCSα subunit. Interestingly, the *Blastocystis* SCSα subunit did provide the CoA binding site essential for the enzyme activity. The refolded WT and gatekeeper mutant *Pf*SCS enzymes were subjected to enzyme kinetics studies. The *Pf*SCS native enzyme was found to be ADP forming (0.36 µm·min^−1^), while a moderate GDP‐forming activity (0.10 µm·min^−1^) was also observed. However, the enzyme kinetics analysis of the recombinantly expressed *Pf*SCS WT‐DY enzyme demonstrated specifically ATP affinity with *K*_mATP_ = 48 µm (Fig. 5A) and no activity with the GTP. The positively charged gatekeeper region of the mutant (GM‐2 KK) emulated the *Blastocystis* SCS WT enzyme in terms of its gatekeeper residues (Lys and Lys). The GM‐2 KK mutant showed a mild decrease in the ATP affinity with *K*_mATP_ = 61 µm (Fig. 5B). To create a negative gatekeeper region, (Tyr→Glu) mutant, GM‐3 DE was constructed, and the enzyme kinetics analysis was carried out. The *K*_mATP_ = 84 µm (Fig. 5C) values again demonstrated the enzyme to be ADP forming exclusively, contrary to the case in *Blastocystis* SCS, where the negative gatekeeper region demonstrated dual‐nucleotide specificity with the introduction of negative gatekeeper residues (Glu and Asp) [7]. To further emulate the sequence‐matched gatekeeper residues from pig SCS, we constructed another mutant GM‐4 ED with Glu and Asp. A similar observation with *K*_mATP_ = 119 µm (Fig. 5D) demonstrated only ATP using the potential of the enzyme. However, we have recorded some insignificant activity with GTP in the case of GM‐3 DE and GM‐4 ED *Pf*SCS enzymes, and thus the *K*_m_ values could not be calculated for GTP.

## Discussion

In the absence of any biochemical studies on *Pf*SCS enzyme with particular focus on its nucleotide specificity, this study stands right with following novel aspects: (a) identifying the corresponding gatekeeper residues from phylogenetically diverse organisms, (b) assessing the substrate specificity of native *Pf*SCS, (c) refolding of recombinantly expressed SCSβ subunits of *P. falciparum* (WT and gatekeeper mutants) and successful refolding in the presence of the *Blastocystis* SCSα subunit, (d) performing enzyme kinetics studies of the refolded enzymes with both nucleotides (ATP and GTP), and (e) determining the effect of the charged gatekeeper residues on the nucleotide specificity. However, it is worth mentioning that with the two separate model systems (*Blastocystis* and *P. falciparum* SCS), where in the case of *Blastocystis* SCS charged gatekeeper residues were able to discriminate between ATP and GTP, the charged gatekeeper residues of *P. falciparum* altered only the binding affinity of ATP, implying that charged gatekeeper residues might be a way of nucleotide discrimination by proteins, but not a general mechanism for ATP versus GTP discrimination.

In an attempt to identify the gatekeeper residues among the phylogenetically diverse organisms using MSA tools, we observed that the most common gatekeeper residues in the ADP‐forming SCS enzymes were Asp and Tyr (*P. falciparum*, *H. sapiens*, *B. taurus*, *M. musculus* and *S. scrofa*), while the GDP‐forming enzymes possessed Glu and Asp residues (*H. sapiens*, *S. scrofa* and *B. taurus*) (Table 1). Interestingly, our previous study [7] has shown that the ADP‐forming *Blastocystis* SCS is unique in having exclusively positively charged gatekeeper residues (Lys and Lys), where alteration of the charges of the gatekeeper region profoundly altered the substrate specificity. However, the *Pf*SCS has distinct gatekeeper residues (Asp and Tyr) matching with others, such as *H. sapiens*, *B. taurus*, *M. musculus* and *S. scrofa*. A peculiar characteristic of the SCS enzyme to have two isoforms in one organism (ADP/GDP‐forming) is worth investigating, with particular focus on the gatekeeper residues. As evident by the MSA analysis, the ADP‐forming SCS enzymes have Asp and Tyr residues, deviating from the GDP‐forming SCS in having Glu and Asp, as gatekeeper residues from the same source. This observation strongly points toward an important role of gatekeeper residues in determining the substrate specificity of the SCS enzyme. However, the analysis of gatekeeper residues in other organisms is beyond the scope of this study.

Enzyme activity of native *Pf*SCS demonstrated the predominantly ADP‐forming activity; however, a moderate GDP‐forming activity was also observed (Fig. 3). It is important to note that the assessment of nucleotide specificity from crude *P. falciparum* lysate is not reliable due to the presence of other parasite proteins, DNA/RNA and nucleotides, metabolites, a variety of other ionic components, etc. Hence we performed the enzyme kinetics analysis with the recombinantly expressed and refolded *Pf*SCS and its mutants. To explore a unique aspect in the refolding process of *Pf*SCS, we used the *Blastocystis* SCSα subunit to refold along with the *Pf*SCSβ subunit. Refolding of the chimeric subunits (SCSα from *Blastocystis* and SCSβ from *P. falciparum*) to a functional enzyme successfully validated that swapping of SCS subunits among different organisms is feasible. Because of the nucleotide binding site in the *Pf*SCSβ subunit, it was possible to investigate the nucleotide specificity of the *Pf*SCS by the chimeric refolded enzyme. The enzyme kinetics studies have demonstrated that in *Pf*SCS, the alteration of the electrostatic properties of the gatekeeper residues did not affect the nucleotide specificity, as it did in our previous serendipitous model enzyme, *Blastocystis* SCS. Surprisingly, the *Blastocystis* SCS enzyme with the positively charged gatekeeper residues favored ATP, whereas with the negatively charged gatekeeper residues, it could use GTP as well, particularly because of the electrostatic interactions with the approaching substrate. This led us to hypothesize that it could be a general mechanism for determining the substrate specificity in other enzymes as well, and it can be further exploited as a novel enzyme engineering approach to alter the substrate specificity. However, in the case of *Pf*SCS, the distinct gatekeeper region as depicted in the electrostatic surfaces models of the WT and various mutants of SCSβ subunits, as compared with the *Blastocystis* SCSβ subunit, was observed. The electrostatic interactions of SCS protein with its approaching substrates (nucleotides) could be masked by other neighboring amino acids and hence could be responsible for a moderate reduction in the ATP affinity of the *Pf*SCS enzyme. However, a detailed structural analysis via molecular modeling and simulation studies could provide a clearer picture of the molecular interactions of the gatekeeper region and the approaching nucleotides in *Pf*SCS. A thorough comparison of the ADP/GDP‐forming isoforms of SCS from the same organism would also be a fruitful attempt in understanding the molecular basis of substrate specificity for enzymes, which can bind to similar substrates, such as ATP/GTP.

## Conclusions

This study concluded that the *Pf*SCS is an ADP‐forming isoform of the SCS enzyme and possesses the gatekeeper residues, which are similar for the ADP‐forming SCS of human, bovine and murine representative organisms. Contrary to our initial assumption that charged gatekeeper residues ‘alone’ could alter the substrate specificity of nucleotide‐binding enzymes such as *Pf*SCS, our experimental data demonstrated only a mere reduction in ATP affinity across all the mutants of *Pf*SCS enzyme. Thus, our study again points out the unanswered question to pinpoint the molecular interactions required for discrimination of similar substrates by the proteins.

## Conflict of interest

The authors declare no conflict of interest.

## Author contributions

KCP and KV conceived and designed the experiments. KV, PS and SV performed the experiments. KCP, KV, RD and NM analyzed the data and wrote the manuscript. All authors reviewed the final version of the manuscript.

## Data Availability

All the data generated from this study are presented in the manuscript.
